# Dietary Consumption of Type 2 Resistant Starch and d‐Fagomine Delays Progression of Metabolic Disturbances in Male Rats on High‐Fat Diet

**DOI:** 10.1002/mnfr.70230

**Published:** 2025-09-09

**Authors:** Bernat Miralles‐Pérez, Sara Ramos‐Romero, María José Charpentier, Vanessa Sánchez‐Martos, Àngels Fortuño‐Mar, Julia Ponomarenko, Susana Amézqueta, David Piñol‐Piñol, Xiang Zhang, Josep Lluís Torres, Marta Romeu

**Affiliations:** ^1^ Facultat De Medicina i Ciències De La Salut Universitat Rovira i Virgili Reus Spain; ^2^ Instituto De Investigaciones Marinas‐Consejo Superior de Investigaciones Científicas (IIM‐CSIC) Vigo Spain; ^3^ Institute For Advanced Chemistry of Catalonia (IQAC‐CSIC) Barcelona Spain; ^4^ Department of Cell Biology Physiology & Immunology Faculty of Biology University of Barcelona Barcelona Spain; ^5^ Nutrition & Food Safety Research Institute (INSA‐UB) Maria De Maeztu Unit of Excellence Santa Coloma de Gramanet Spain; ^6^ Eldine Patología Tarragona Spain; ^7^ Centre For Genomic Regulation (CRG) The Barcelona Institute of Science and Technology Barcelona Spain; ^8^ Universitat Pompeu Fabra (UPF) Barcelona Spain; ^9^ Departament D'enginyeria Química i Química Analítica Universitat De Barcelona Spain; ^10^ Department of Chemistry University of Louisville Louisville Kentucky USA

**Keywords:** atherogenic dyslipidemia, fiber, iminosugar, oxidative stress, prediabetes

## Abstract

High‐fat (HF) diets contribute to obesity, insulin resistance, fatty liver, gut microbiota dysbiosis, oxidative stress, and low‐grade chronic inflammation. This study evaluated the preventive effects of dietary Type 2 resistant starch (RS2) from high‐amylose maize and low‐dose d‐fagomine (FG) from buckwheat on these metabolic disturbances. Male Wistar‐Kyoto rats (9–10 weeks old) were assigned to four diet groups for 10 weeks: standard (STD) diet, HF diet (45% kcal from fat), HF + RS diet (15% RS2), and HF + FG diet (0.1% FG). Body characteristics, metabolic parameters, oxidative stress, gut microbiota, short‐chain fatty acids (SCFAs), and eicosanoids were analyzed. Both HF + RS and HF + FG diets reduced perigonadal fat, plasma triacylglycerols, and oxidative stress. HF + RS diet improved glucose tolerance without significantly affecting insulin sensitivity, while HF + FG diet showed a tendency for improvement at later stages. Additionally, HF + RS diet showed greater beneficial effects on body weight and liver steatosis than HF + FG diet, likely due to gut microbiota and SCFA modulation. RS2 exerted stronger metabolic effects than FG under HF diet conditions, suggesting its greater potential in mitigating obesity‐related complications. FG effects may require longer exposure to manifest.

## Graphical abstract

This study compared the preventive effects of dietary resistant starch (RS2) from maize and d‐fagomine (FG) from buckwheat on cardiometabolic risk factors in high‐fat (HF) diet‐fed rats. Wistar‐Kyoto rats (*n* = 11–12/group) received STD, HF, HF + RS, or HF + FG diets for 10 weeks. HF + RS and HF + FG reduced perigonadal fat, triacylglycerols, and oxidative stress but did not improve insulin sensitivity. HF + RS had greater benefits on weight, glucose tolerance, and liver steatosis, likely due to gut microbiota modulation.

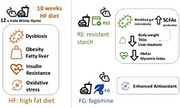

**Abbreviations**
4‐HAE: 4‐hydroxyalkenal
ALT: alanine aminotransferase
AMPK: adenosine monophosphate‐activated protein kinase
AST: aspartate aminotransferase
AUC: area under the curve
CAT: catalase
FG: d‐fagomine
GPx: glutathione peroxidase
GR: glutathione reductase
GSH: glutathione reduced
GSSG: glutathione oxidized
HbA1c: glycated hemoglobin
HF: high‐fat
HF+FG: high‐fat + 0.1% of buckwheat d‐fagomine
HF+RS: high‐fat + 15% of high‐amylose maize resistant starch Type 2
HOMA‐IR: Homeostatic Model Assessment of Insulin Resistance
MDA: malondialdehyde
NAFLD: non‐alcoholic fatty liver disease
NO: nitric oxide
${\mathrm{NO}}_{2}^{-}$
: nitrite anion
NOX: NADPH oxidase
OGTT: oral glucose tolerance test
PAT: perigonadal white adipose tissue
ROS: reactive oxygen species
RS: resistant starch
RS2: Type 2 resistant starch
SOD: superoxide dismutase
STD: standard
TAG: triacylglycerol
TBARS: thiobarbituric acid‐reactive substance
WKY: Wistar‐Kyoto
ZDF: Zucker diabetic fatty

## 1 Introduction

Western diet, rich in energy‐dense foods and saturated fats, combined with low content of fiber, contributes to the development of obesity, non‐alcoholic fatty liver disease (NAFLD), atherogenic dyslipidemia, hypertension, impaired glucose regulation, and Type 2 diabetes mellitus, among other cardiometabolic disturbances [1, 2]. This type of diet can also promote chronic oxidative stress, low‐grade chronic inflammation, and gut microbiota dysbiosis [3, 4]. Oxidative stress occurs when the production of oxidants, such as reactive oxygen species (ROS) and reactive nitrogen species, exceeds the capacity of the body's antioxidant defenses. This imbalance can lead to impaired redox signaling and/or cause oxidative damage to biomolecules [5]. Oxidative stress is closely linked to inflammation, which is related to increased production of ROS, cytokines, chemokines, adhesion molecules, and eicosanoids. Of these, eicosanoids are a type of oxygenated lipid mediators capable to influence cellular homeostasis. Under oxidative stress and inflammatory stimuli, a variety of eicosanoids can be generated from 20‐carbon polyunsaturated fatty acids (PUFAs) of the n‐3 and n‐6 series via non‐enzymatic and enzymatic pathways, showing those eicosanoids derived from n‐6 PUFAs an overall proinflammatory character [6].

Diet plays a crucial role in shaping the composition and function of the gut microbiota, with various dietary patterns leading to distinct changes in microbial communities [7]. The Western diet is linked to reduced microbial diversity and a rise in harmful bacteria. In contrast, fiber‐rich diets, like the Mediterranean diet, which also includes healthy fats and polyphenols, support the growth of beneficial bacteria and increase microbial diversity [3]. An altered gut microbiota can impact metabolic health by influencing bacterial metabolite production (e.g., short‐chain fatty acids [SCFAs]), as well as by promoting low‐grade chronic inflammation and elevated oxidative stress levels [8].

Starch, a glucose polymer used by plants for energy storage, is broken down by enzymes in animals' digestive systems into glucose units for absorption. Its digestibility is classified into three types: rapidly digestible starch, slowly digestible starch, and resistant starch (RS) [9]. RS is considered a form of dietary fiber because it reaches the large intestine almost intact, where it is then digested and metabolized by the resident microorganisms [9]. RS has been classified into five major groups based on its accessibility to digestive enzymes in the human intestine [9]. Furthermore, based on gut microbiota function, other new classification of RS has been proposed according to its fermentation rate (rapidly fermentable, slowly fermentable, or nonfermentable), according to the type of metabolites produced (butyrate, propionate, or acetate promoters), according to the number of bacterial groups capable of processing them (highly specific or less specific), and according to the preferred phylum they promote (Bacteroidetes, Firmicutes, or Actinobacteria promoters) [10].

Regular food starch is quickly broken down into glucose, causing blood glucose spikes, insulin secretion, and potential hypoglycemia. Repeated cycles of these highs and lows can contribute to obesity, which is a key factor in the development of insulin resistance and Type 2 diabetes [9]. In contrast, RS may be an effective strategy for preventing or delaying the onset of insulin resistance and related disorders by reducing glycemic index and caloric value of food as well as modulating gut microbiota and production of putatively beneficial SCFAs [9, 11]. The dietary intake of RS in western countries is 3–9 g per day while a higher consumption (∼15 g/day) is considered adequate for the promotion of gastrointestinal health and the improvement of insulin sensitivity [12, 13]. Type 2 resistant starch (RS2) is a naturally occurring form of starch with a granular structure that is inaccessible to digestive enzymes and amylases [11]. Unlike other RSs, which require additional processing or interactions with other dietary components, RS2 naturally forms dense, amylose‐rich granules that are resistant to digestion [11]. It is present in high‐amylose maize, raw potatoes, and green bananas [9], and is commonly used as a functional ingredient [14], being classified as a slowly fermentable starch [15]. Consumption of RS2 from high‐amylose maize improves insulin sensitivity in men with obesity at daily doses of 15–30 g [16], and promotes weight loss in overweight or obesity conditions and decreases liver triacylglycerols (TAGs) in people with NAFLD at a daily dose of 40 g [17, 18]. In previous rodent studies, RS has been supplemented in the feed at levels ranging from 10% to 30% to evaluate its physiological effects [19]. The relatively high amounts of RS needed for eliciting its functional effects may be hard to accept for the average consumer. Bread containing up to 20% high‐amylose maize RS2 maintained its quality (specific volume, crumb porosity, and texture) and acceptability, whereas bread with 30% RS2 presented altered properties and low acceptability [14].

Another molecule with potential fiber‐like effects is d‐fagomine (FG, (2R, 3R, 4R)‐2‐hydroxymethylpiperidine‐3,4‐diol; 1,2‐dideoxynojirimycin) [20, 21]. FG, first isolated from buckwheat (*Fagopyrum esculentum* Moench) [22], is a glucose analog because the spatial configurations of the hydroxyl groups at Positions 3, 4, and 6 in its saturated six‐membered ring match those in d‐glucose. Unlike d‐glucose, FG is resistant to metabolic degradation because it includes a nitrogen atom in the position of the anomeric carbon [23]. FG is a mild glycosidase inhibitor in vitro and it is particularly effective at reducing post‐prandial blood glucose concentration when administered together with either sucrose or starch [24]. Moreover, FG has been proved to modify the populations of some gut microorganisms, probably through an antiadhesive activity related to its structural similarity with glucose and mannose [20, 24, 25]. Both activities (glycosidase inhibition and bacterial adhesion/antiadhesion) may be behind the in vivo effect of FG against different cardiometabolic risk factors [25, 26].

For these reasons, we compared here the potential preventive effects of the high‐amylose maize RS2 (15% by weight of feed) and the FG (0.1%; minimal active dose) on the onset and/or development of cardiometabolic disorders in a rat model of diet‐induced obesity and prediabetes.

## 2 Experimental Section

### 2.1 Animal Experiment and Diets

A cohort of 47 male Wistar‐Kyoto (WKY/NHsd) rats, obtained from Envigo (currently Inotiv, Indianapolis, IN, USA), was housed in groups of three per cage under controlled environmental conditions: 60% humidity, a temperature of 22 ± 2°C, and a 12‐h light/dark cycle. The WKY rats were chosen as a model for diet‐induced obesity and insulin resistance due to their tendency to develop impaired glucose tolerance more rapidly than other strains, such as Sprague‐Dawley rats. To minimize circadian rhythm interference, all procedures were conducted in the morning.

The animal feeding experiment lasted for 10‐week and a randomized allocation strategy was employed to assign animals, aged 9–10 weeks, into four groups (*n* = 11–12 per group): a standard (STD) group fed a standard diet (2014 Teklad Global 14% Protein [Envigo (currently Inotiv), Indianapolis, IN, USA]), a high‐fat (HF) group fed an HF diet (TD.08811, 45% kcal from fat [Envigo (currently Inotiv), Indianapolis, IN, USA]), a group fed the HF diet with 15% of high‐amylose maize RS2 by feed weight (high‐fat + 15% of high‐amylose maize resistant starch Type 2 [HF+RS]), and a group fed the HF diet with 0.1% of buckwheat FG by feed weight (high‐fat + 0.1% of buckwheat d‐fagomine [HF+FG]). Detailed diet compositions are outlined in Table S1. RS2 (HYLON VII PCR) was provided by Ingredion (Hamburg, Germany). FG (>98% purity) was produced by Bioglane SLNE (Barcelona, Spain) and generously provided by Taihua Shouyue (HK) International Co. Ltd. (Hong Kong, China). The 15% of high‐amylose maize RS2 dosage reflects the highest amount used in prior studies in rats, indicating that beneficial metabolic effects can be observed at this range while maintaining good tolerability [27]. The 0.1% FG dose corresponds to the minimum active dose reported (2 mg/g carbohydrate) [24].

All experimental animal protocols were executed in strict conformity with the European Union guidelines for the ethical care and use of laboratory animals (Directive 2010/63/EU). Formal authorization for animal experimentation was granted by the Catalan authorities under license number 10090 and received explicit approval from the Bioethical Issues Subcommittee of the Spanish National Research Council (CSIC).

### 2.2 Reporting Dose and Administration Details

Rats were fed ad libitum with their respective experimental diets for 10 weeks. The diet was administered orally through standard chow feeding, ensuring voluntary consumption. The frequency of administration was daily intake, simulating a long‐term dietary intervention.

Previous research suggests RS2 and FG are generally well tolerated, and are beneficial for gut health.

### 2.3 Characteristics of Rats and Sample Collection

Body weight and food intake was monitored weekly. Energy intake was calculated by estimating metabolizable energy using the Atwater factors, assigning 4 kcal/g to protein, 9 kcal/g to fat, and 4 kcal/g to available carbohydrates except for RS, which used a factor of 2.8 kcal/g RS [28]. During Weeks 5 and 6, the rats were placed individually in metabolic cages overnight to record individual data of feed and water intakes and feces and urine excretions. Fecal samples were collected during Weeks 6 and 9 through abdominal massage, then promptly frozen and stored at −80°C. At Weeks 6 and 10 of the study, blood samples were taken from the saphenous vein of fasted rats. Plasma was separated by centrifugation at 1300 × *g* for 5 min at 4°C and stored at −80°C.

During Week 10, the rats were fasted overnight before being anesthetized intraperitoneally with a combination of ketamine (Merial Laboratorios, Barcelona, Spain) and xylazine (Química Farmacéutica, Barcelona, Spain) at doses of 80 and 10 mg/kg body weight, respectively. Following anesthesia, peritoneal macrophages were collected by injecting 40 mL ice‐cold sterile PBS (pH 7.2) into the peritoneal cavity. After abdominal massage, cell suspension was aspirated, centrifuged, and resuspended in cold DMEM+GlutaMAX media (Invitrogen, Paisley, UK) containing 10% fetal bovine serum (FBS; PAA, Pasching, Austria), and 100 IU/mL streptomycin–penicillin (Sigma; DMEM+G‐FBS).

Blood was collected by cardiac puncture while the heart was still beating, ensuring exsanguination. Plasma was promptly separated and stored at −80°C. Tissues, including the cecum, the liver, and perigonadal white adipose tissue (PAT), were removed, weighed, and immediately frozen in liquid nitrogen and then stored at −80°C. Portions of the liver and PAT were fixed in 4% formaldehyde for 24 h.

### 2.4 Glycemic Status, Lipid Profile, and Transaminases

Oral glucose tolerance tests (OGTTs) were conducted during Weeks 4 and 8 on fasted animals. Blood glucose was measured at 0, 15, 30, 45, 60, 90, and 120 min after administering oral glucose (1 g/kg body weight), using the enzyme electrode method with an Ascensia ELITE XL blood glucose meter (Bayer Consumer Care AG, Basel, Switzerland).

Blood glucose and plasma insulin were measured at Weeks 6 and 10 in fasted animals. Blood glucose was determined using the same enzyme electrode method described above. Plasma insulin was measured using a rat/mouse ELISA kit (Millipore Corporation, Billerica, MA, USA). The Homeostatic Model Assessment of Insulin Resistance (HOMA‐IR) index, an indicator of insulin resistance, was calculated using the formula: fasting insulin (µU/mL) × fasting glucose (mmol/L)/22.5 [29]. Insulin units (IUs) were converted using the factor 1 IU = 0.0347 mg of insulin. Glycated hemoglobin (HbA1c) was measured by means of spectrophotometry using commercial kits (Spinreact, Girona, Spain) in a COBAS MIRA autoanalyzer (Roche Diagnostics System, Madrid, Spain) at Week 10 in fasted animals. At Weeks 6 and 10, plasma TAGs and cholesterol were also analyzed in fasted animals, and at Week 10, low‐density lipoprotein cholesterol (LDLc), high‐density lipoprotein cholesterol (HDLc), aspartate aminotransferase (AST), and alanine aminotransferase (ALT) were assessed using the same methodology.

### 2.5 DNA Extraction and Sequencing of Fecal Microbiota

DNA extraction and sequencing were carried out as previously described [30]. Briefly, total DNA was extracted from feces collected at Week 9 using using the QIAamp DNA Stool Mini Kit (QIAGEN, Hilden, Germany) and measured in a Nanodrop 8000 Spectrophotometer (ThermoScientific, Waltham, MA, USA). Then, samples of DNA were diluted for amplification of the V3–V4 regions of the 16S ribosomal RNA (rRNA) gene using a limited cycle PCR with the following universal primers:

Forward primer: 5′ TCG TCG GCA GCG TCA GAT GTG TAT AAG AGA CAG CCT ACG GGN GGC WGC AG

Reverse primer: 5′ GTC TCG TGG GCT CGG AGA TGT GTA TAA GAG ACA GGA CTA CHV GGG TAT CTA ATC C

Two DNA samples derived from bacterial mock communities obtained from ZymoBIOMICS were used as control for sequencing and downstream procedures. Sequencing was performed on an Illumina MiSeq, with 2 × 300 bp reads using v3 chemistry with a loading concentration of 10 pM, at the Genomics Unit of the Centre for Genomic Regulation, Barcelona.

### 2.6 Sequencing Analysis

The R package Phyloseq (version 1.36.0) was used for analyzing of the 16S rRNA operational taxonomic unit counts in R version 4.1.0 [31]. The Wald test in DESeq2 package (version 1.32.0) was used for evaluation of statistical significance of the relative abundances of taxa [32]. Furthermore, indexes to estimate within‐sample α‐diversity, including richness (i.e., Chao1 index), evenness (i.e., Pielou's and Bulla's indexes), dominance (i.e., Simpson's, Berger–Parker's and Relative indexes), rarity (i.e., log modulo skewness index), and diversity (i.e., Inverse Simpson's and Gini's indexes), were calculated by means of the R package microbiome (version 1.13.12) [33]. The R packages Phyloseq and vegan (version 2.5.7) were used for calculation of between‐sample β‐diversity (i.e., principal coordinate analysis of unweighted and weighted Unifrac distance) and its statistical significance between the groups (Permanova analysis with Adonis) [34].

### 2.7 Short Chain Fatty Acids

SCFAs were measured in fecal samples after 6 and 9 weeks of intervention, and in cecal content at the end of the study (Week 10) using gas chromatography with flame ionization detection as previously described [30].

### 2.8 Histological Analysis

Formalin‐fixed PAT and liver samples were processed, and the hematoxylin‐eosin‐stained sections were examined by a single‐blinded pathologist as described elsewhere [35] and graded as presented in Tables 3 and 4.

### 2.9 Oxidative Stress

Nitrite anion (${\mathrm{NO}}_{2}^{-}$), the stable by‐product of nitric oxide (NO), was spectrophotometrically measured in lyophilized urine, obtained during Weeks 5–6, by a modification of the Griess reaction as previously described [36].

Isolation of peritoneal macrophages and subsequent measurement of basal production of intracellular ROS by means of dichlorofluorescein assay were performed at Week 10 as previously described [37]. Areas under the curve (AUCs) were calculated using the Trapezium method (fluorescence unit/2.5 × 10^4^ cells per 100 mL per 120 min).

Endogenous antioxidants, including superoxide dismutase (SOD), catalase (CAT), glutathione peroxidase (GPx), and glutathione reductase (GR), were determined in erythrocytes, PAT, and liver as previously described [38, 39, 40]. Glutathione, both reduced (glutathione reduced [GSH]) and oxidized (glutathione oxidized [GSSG]) forms, was quantified in plasma, PAT, and liver following established protocols [41].

Lipid peroxidation by‐products were evaluated using thiobarbituric acid‐reactive substances (TBARSs) and malondialdehyde (MDA) plus 4‐hydroxyalkenal (4‐HAE) assays. TBARS in plasma, erythrocytes, and PAT were measured as described elsewhere [42] with some modifications [43]. MDA+4‐HAE in the liver was measured as previously described [44].

To normalize parameters of oxidative stress, blood hemoglobin (Hb) and tissue protein were measured as described elsewhere [45, 46].

### 2.10 Eicosanoids

Liver eicosanoids were measured as previously described [47] with minor modifications [30] using an ACQUITY UPLC system coupled to a Xevo TQ‐S micro mass spectrometer equipped with a BEH C18 column (1.7 µm, 2.1 × 100 mm), which was protected by a Vanguard precolumn (1.7 µm, 2.1 × 5 mm) and maintained at 45°C (Waters, Milford, MA, USA). The mobile phases were (A) 0.1% acetic acid and (B) an acetonitrile/isopropanol mixture (90:10, v/v), with a flow rate of 0.6 mL/min. The elution gradient began at 25% B, increased linearly from 25% to 95% B from 1.00 to 8.00 min, remained at 95% B for 0.50 min, and reconditioned from 8.51 to 10.00 min. Ten microliters of sample were injected. The analysis was performed using a multiple reaction monitoring method in negative mode (Table S2). After normalization to the corresponding internal standards, eicosanoids were quantified using the corresponding external standard curves (Cayman Chemical, Ann Arbor, MI, USA).

### 2.11 Statistical Analysis

The statistical analysis was performed using SPSS v.26 software (IBM, Chicago, IL, USA). The normal distributions of the data were evaluated with the Shapiro–Wilk test, and statistical significance was determined by one‐way ANOVA and the Tukey multiple‐comparisons test if data was normally distributed or by Kruskal–Wallis test and the Mann–Whitney *U* test for nonparametric data. The results are expressed as means with their standard errors (standard error of the mean [SEM]), except for histological results which are presented in frequencies (%) or median and 25th–75th percentiles. Differences were considered statistically significant when *p* value ≤ 0.05, and were considered to indicate a tendency when *p* value ≤ 0.1.

## 3 Results

### 3.1 Characteristics of Rats

**FIGURE 1:**
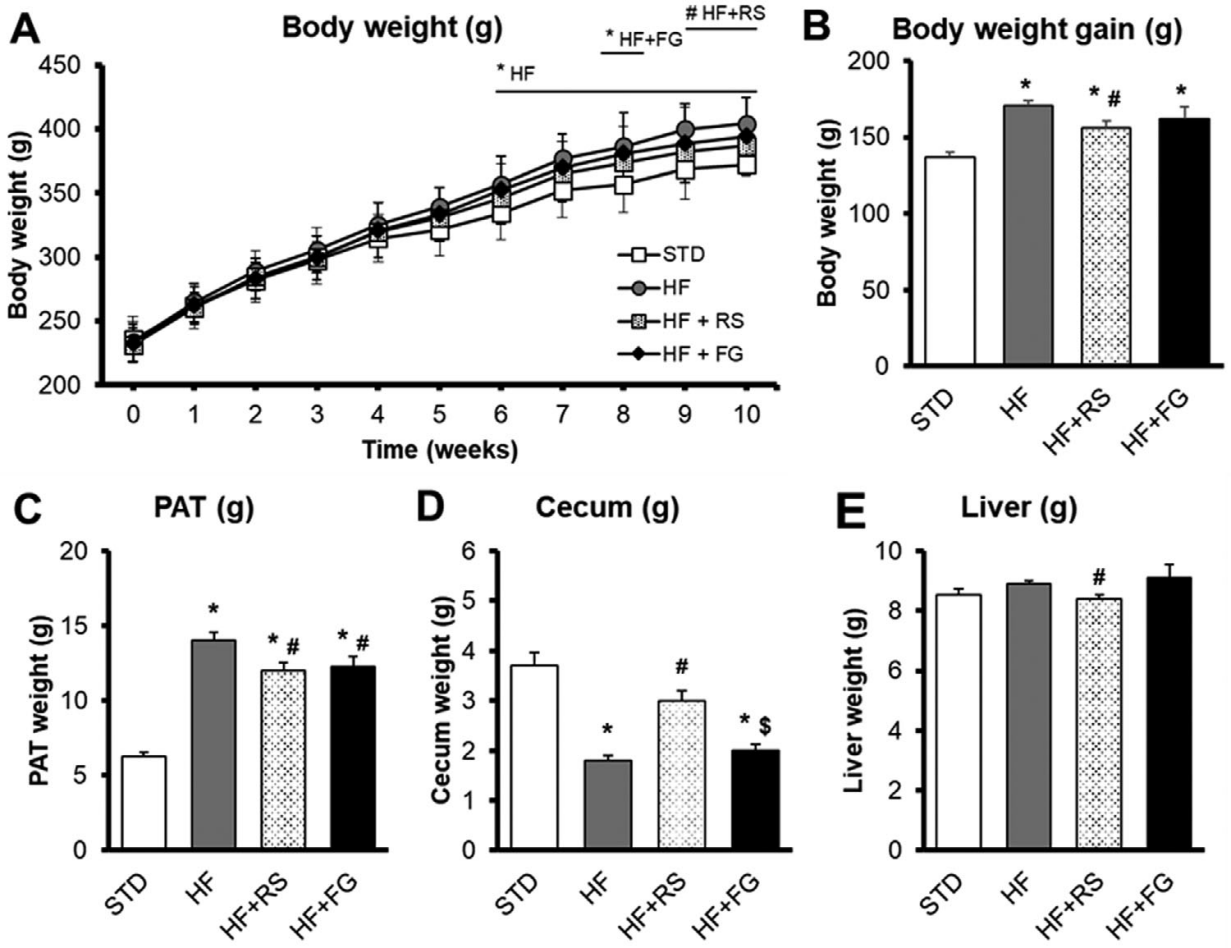
Characteristics of rats. (A) Body weight evolution for 10 weeks of nutritional intervention, (B) Body weight gain, (C) Perigonadal adipose tissue weight, (D) Cecum weight, and (E) Liver weight. Values are expressed as means with their SEM; *n* = 11–12 per group. STD, rats fed a standard diet; HF, rats fed a high‐fat diet; HF+RS, rats fed a high‐fat diet with 15% of high‐amylose maize resistant starch Type 2 by feed weight; HF+FG, rats fed a high‐fat diet with 0.1% of buckwheat d‐fagomine by feed weight. *p*‐values were calculated using the one‐way ANOVA and the Tukey multiple‐comparisons test or by the nonparametric Kruskal–Wallis test and the Mann–Whitney *U* test. * *p*‐value ≤ 0.05 versus STD (HF group during Weeks 6–10, whereas HF+FG group only at Week 8); # *p*‐value ≤ 0.05 versus HF; and $ *p*‐value ≤ 0.05 versus HF+RS. ANOVA, analysis of variance; FG, d‐fagomine; HF, high‐fat; RS, resistant starch; SEM, standard error of the mean; STD, standard.

Body weight in the HF group was higher than those values observed in the STD group after 6 weeks of intervention and maintained until the end (Week 10), while the HF+RS showed similar body weight throughout the study than the STD group and the HF+FG group showed a punctual increased body weight only at Week 8 (Figure 1A). So, the HF+RS group showed attenuation of body weight gain compared to the HF group (Figure 1B). The three HF groups showed reduced feed intake compared to the STD group, while their energy intake remained higher throughout the experiment. In Week 5, samples collected from the metabolic cages revealed that water intake and urine excretion were similar across all groups. However, the HF and HF+FG groups presented lower amount of feces collected than the STD group. In contrast, the HF+RS group showed values closer to the STD group, with a trend toward significance (*p* value = 0.061; data not shown).

For organs, PAT weight was higher in the three HF groups than the values found in the STD group (Figure 1C). Nevertheless, the HF+RS and HF+FG groups showed decreased PAT weight compared to the HF group. The HF and HF+FG groups showed lower cecum weight than the STD and HF+RS groups (Figure 1D). The HF+RS group showed lower liver weight than the values observed in the HF group and tented to decrease liver weight compared to the HF+FG group (*p* value = 0.058; Figure 1E).

### 3.2 Glycemic Status

**FIGURE 2:**
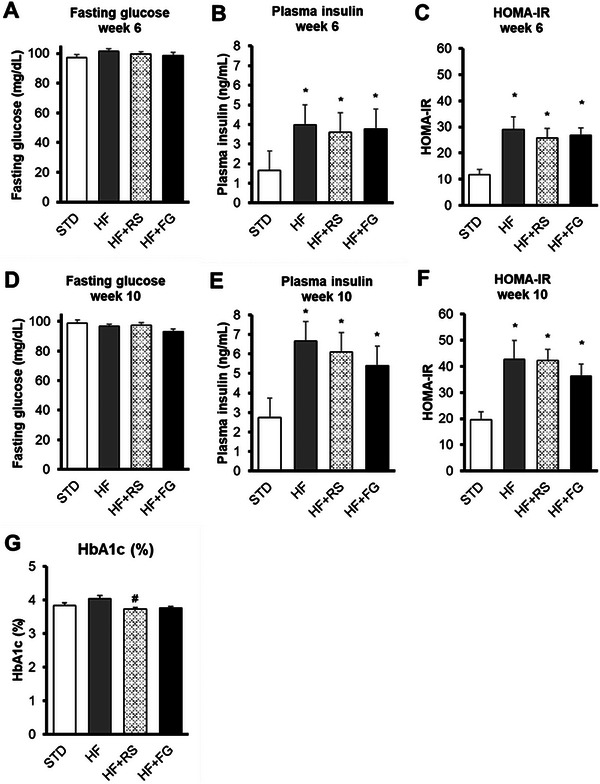
Glucose homeostasis in fasted rats. (A) Blood glucose at Week 6. (B) Plasma insulin at Week 6. (C) HOMA‐IR at Week 6. (D) Blood glucose at Week 10. (E) Plasma insulin at Week 10. (F) HOMA‐IR at Week 10. (G) Blood glycated hemoglobin (HbA1c) at Week 10. Values are expressed as means with their SEM; *n* = 11–12 per group. STD, rats fed a standard diet; HF, rats fed a high‐fat diet; HF+RS, rats fed an HF diet with 15% of high‐amylose maize resistant starch Type 2 by feed weight; HF+FG, rats fed an HF diet with 0.1% of buckwheat d‐fagomine by feed weight; HOMA‐IR, HOMA‐IR (insulin [µU/mL] × glucose [mmol/L]/22.5). *p*‐values were calculated using the one‐way ANOVA and the Tukey multiple‐comparisons test or by the nonparametric Kruskal–Wallis test and the Mann–Whitney *U* test. *p‐value ≤ 0.05 versus STD; and #*p*‐value ≤ 0.05 versus HF. ANOVA, analysis of variance; FG, d‐fagomine; HF, high‐fat; HOMA‐IR, Homeostatic Assessment Model of Insulin Resistance; RS, resistant starch; SEM, standard error of the mean; STD, standard.

Fasting plasma insulin (Figure 2B, E) and HOMA‐IR (Figure 2C, F) were higher in the three HF groups than in the STD group at Weeks 6 and 10. Although fasting blood glucose (Figure 2A, D) was similar among the groups during the study, the HF+RS group showed lower values of HbA1c than those values observed in the HF group, and the HF+FG group showed a tendency for lower values (*p* value = 0.054) at the end of the study (Figure 2G).

**FIGURE 3:**
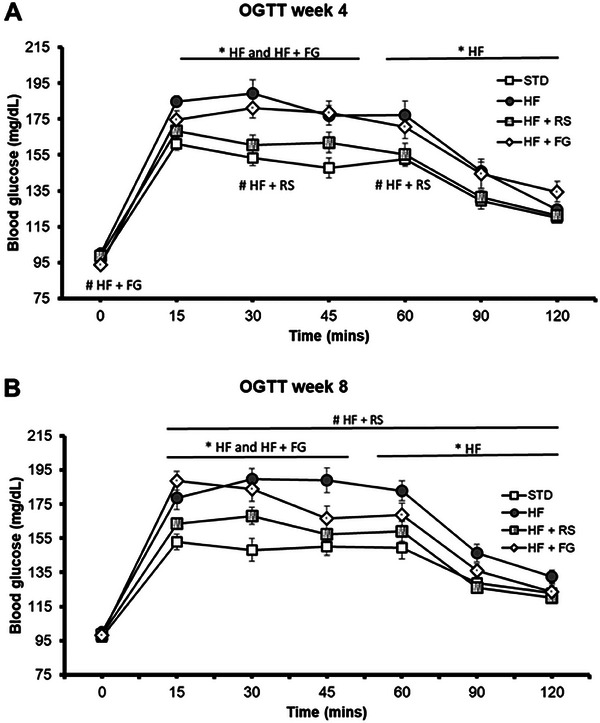
(A) OGTTs during Week 4. (B) OGTTs during Week 8. Values are expressed as means with their SEM; *n* = 11–12 per group. STD, rats fed a standard diet; HF, rats fed a high‐fat diet; HF+RS, rats fed a high‐fat diet with 15% of high‐amylose maize resistant starch type 2 by feed weight; HF+FG, rats fed a high‐fat diet with 0.1% of buckwheat d‐fagomine by feed weight. *p* values were calculated using the one‐way ANOVA and the Tukey multiple‐comparisons test or by the nonparametric Kruskal–Wallis test and the Mann–Whitney *U* test. *p‐value ≤ 0.05 versus STD; #*p*‐value ≤ 0.05 versus HF; and $*p*‐value ≤ 0.05 versus HF+RS. ANOVA, analysis of variance; FG, d‐fagomine; HF, high‐fat; RS, resistant starch; OGTT, oral glucose tolerance test; SEM, standard error of the mean; STD, standard.

Postprandial blood glucose (OGTTs) was different between the STD and HF groups from 15 to 60 min after glucose administration at Weeks 4 and 8 (Figure 3A, B), indicating impaired glucose tolerance in the HF group. The curves corresponding to the HF+RS and STD groups were similar at both Weeks 4 and 8. The curves corresponding to the HF and HF+FG groups were similar at Week 4 (Figure 3A) and higher than those curves of the STD group at the central points (30 and 45 min). At Week 8, the HF+FG group recorded a lower glucose value than that corresponding to the HF group 45 min after the glucose load (Figure 3B), modulating the typical plateau associated with the insulin resistance status compared to the STD group.

### 3.3 Plasma Lipid Profile and Transaminases

At Week 6, plasma TAGs in the HF and HF +FG groups were increased compared to those values found in the STD group (Table 1), whereas the HF+RS group presented decreased TAGs compared to the HF group. These differences were also observed at the end of the study (Week 10) but the HF+FG group showed decreased TAGs compared to the HF group, although it remained higher than in the STD group.

**TABLE 1:** Lipid profile and transaminases in plasma of rats.

	STD (*n* = 12)	HF (*n* = 12)	HF+RS (*n* = 11)	HF+FG (*n* = 12)
TAGs (mmol/L), Week 6	1.09 ± 0.06	1.54 ± 0.06^*^	1.20 ± 0.04^**^	1.33 ± 0.07^*^
TAGs (mmol/L), Week 10	0.58 ± 0.03	0.93 ± 0.04^*^	0.69 ± 0.03^**^	0.78 ± 0.03^*,**^
Cholesterol (mmol/L), Week 6	3.92 ± 0.04	3.65 ± 0.06^*^	3.47 ± 0.06^*,**^	3.77 ± 0.10^*^
Cholesterol (mmol/L), Week 10	2.92 ± 0.10	2.99 ± 0.05	2.99 ± 0.06	3.01 ± 0.05
LDLc (mmol/L)	0.39 ± 0.03	0.39 ± 0.01	0.47 ± 0.01^*,**^	0.46 ± 0.01^*,**^
HDLc (mmol/L)	2.04 ± 0.10	2.30 ± 0.03	2.52 ± 0.05^*,**^	2.54 ± 0.06^*,**^
Ratio LDLc/HDLc	0.19 ± 0.01	0.17 ± 0.01^*^	0.19 ± 0.01	0.18 ± 0.01
Ratio TAGs/HDLc	0.29 ± 0.01	0.41 ± 0.02^*^	0.28 ± 0.01	0.31 ± 0.01
AST (U/L)	148 ± 17	159 ± 9	184 ± 15	157 ± 12
ALT (U/L)	42 ± 5	39 ± 3.5	39 ± 5	39 ± 1
Ratio AST/ALT	4.4 ± 1	4.2 ± 0.3	6.0 ± 1	4.0 ± 0.8

*Note*: Values are expressed as mean with their SEM. The number of animals per group is indicated in the table header. *p* values were calculated using the one‐way ANOVA and the Tukey multiple‐comparisons test or by the nonparametric Kruskal–Wallis test and the Mann–Whitney *U* test. *p‐value ≤ 0.05 versus STD; #*p*‐value ≤ 0.05 versus HF; and $*p*‐value ≤ 0.05 versus HF+RS.

Abbreviations: ALT, alanine aminotransferase; AST, aspartate aminotransferase; HDL, high‐density lipoprotein cholesterol; HF, rats fed a high‐fat diet; HF+FG, rats fed a high‐fat diet with 0.1% of buckwheat d‐fagomine by feed weight; HF+RS, rats fed a high‐fat diet with 15% of high‐amylose maize resistant starch type 2 by feed weight; LDL, low‐density lipoprotein cholesterol; STD, rats fed a standard diet; TAG, triacylglycerol.

At Week 6, plasma cholesterol in the three HF groups was decreased compared to those values found in the STD group (Table 1). The HF+RS group also showed lower cholesterol than that observed in the STD group (Table 1). Despite the lack of differences in total cholesterol at the end of the study (Week 10) among the groups, when compared to the STD diet, the HF diet affected values of LDLc/HDLc and TAGs/HDLc ratios, which were normalized by the inclusion of either RS or FG into the HF diet.

No statistically significant differences were found in either transaminases or corresponding ratio in plasma among the groups (Table 1).

### 3.4 Fecal Microbiota

The within‐sample α‐diversity indexes differed between the HF and STD diet‐fed rats at both phylum and genus levels. The HF group showed increased evenness and decreased rarity, especially at the phylum level compared to the STD group (*p* value ≤ 0.05). Although the HF+FG group showed a similar α‐diversity pattern to that observed in the HF group, the HF+RS group reverted the increase in evenness and the decrease in rarity promoted by the HF diet and showed lower richness, lower diversity, and higher dominance, especially at genus level (*p* value ≤ 0.05). There were no statistically significant differences in between‐sample β‐diversity either at the phylum or genus levels among the groups.

In particular, the HF group showed higher Proteobacteria phylum together with increased *Blautia*, *Clostridium* XlVa, *Desulfovibrio*, *Escherichia/Shigella*, and *Rothia* genera but decreased unclassified *Candidatus Saccharibacteria*, *Clostridium* IV, unclassified *Muribaculaceae*, and *Prevotella* genera than the STD group (*p* value ≤ 0.05). When compared to the HF group, the HF+RS group reverted the increase in Proteobacteria phylum, while increasing Bacteroidetes and Firmicutes phyla (*p* value ≤ 0.05). The effect of RS on Proteobacteria phylum could be achieved by decreasing *Desulfovibrio* and *Escherichia*/*Shigella* genera (*p* value ≤ 0.05). Furthermore, the HF+RS group prevented the decrease in the unclassified *Muribaculaceae* genus from the Bacteroidetes phylum. Nevertheless, the HF+RS group decreased *Bacteroides* and *Parabacteroides* genera (*p* value ≤ 0.05). At Firmicutes phylum, the HF+RS group increased unclassified *Clostridiales*, unclassified *Ruminococcaceae* and *Turicibacter* genera, but decreased unclassified *Firmicutes* and *Lactococcus* (*p* value ≤ 0.05). Unlike the HF+RS group, the HF+FG group showed a similar composition of gut microbiota to that observed in the HF group.

### 3.5 Short Chain Fatty Acids in Fecal and Cecal Samples

In fecal samples, at Week 6, total SCFAs (Table 2) were decreased in the HF and HF+FG groups compared to the STD group, while it was higher in the HF+RS group than in the HF group. In particular, acetic and propionic acids were lower in the HF and HF+FG groups than in the HF+RS and STD groups. Although no statistically significant differences were found in isobutyric and isovaleric acids between the HF and STD groups, these values were especially lower in the HF+RS and HF+FG groups than those observed in the STD group. No statistically significant differences were observed among the groups for butyric and valeric acids. At Week 9, total SCFAs were lower in the three HF diet groups than in the STD group. Acetic and propionic acids were lower in the HF and HF+FG groups than in the STD group, while the HF+RS group showed attenuation of the decrease in these SCFAs. The other SCFAs were decreased in the three HF groups compared to the STD group. At the end of the study, total SCFAs (Table 2) in cecal samples were higher in the HF+RS group than in the other groups, mainly due to increased amounts of acetic, propionic, and butyric acids. Isobutyric and isovaleric acids were lower in the HF and HF+RS groups than in the STD group. Furthermore, the HF+RS group showed decreased isovaleric acid compared to the HF+FG group. Values of valeric acid were similar among the groups.

**TABLE 2:** Short‐chain fatty acids in feces after 6 and 9 weeks of intervention and in cecal content (dry matter).

		STD (*n* = 12)	HF (*n* = 12)	HF+RS (*n* = 11)	HF+FG (*n* = 12)
Acetic acid (mmol/kg)	Feces Week 6	177 ± 32	63 ± 9^*^	164 ± 27^**^	66 ± 10^*,***^
	Feces Week 9	133 ± 16	29 ± 3^*^	46 ± 6	27 ± 3^*^
	Cecal content	108 ± 8	111 ± 8	135 ± 6^*^	107 ± 1^***^
Propionic acid (mmol/kg)	Feces Week 6	17.9 ± 3.3	4.3 ± 1.0^*^	19.1 ± 4.3^**^	3.4 ± 0.7^*,***^
	Feces Week 9	14.0 ± 2.2	2.2 ± 0.5^*^	4.4 ± 0.6	2.2 ± 0.3^*^
	Cecal content	28 ± 2	29 ± 2	39 ± 2^*,**^	33 ± 2
Isobutyric acid (mmol/kg)	Feces Week 6	1.7 ± 0.3	0.9 ± 0.3	0.4 ± 0.1^*^	0.5 ± 0.1^*^
	Feces Week 9	1.3 ± 0.2	0.2 ± 0.1^*^	0.1 ± 0.1^*^	0.3 ± 0.1^*^
	Cecal content	4.1 ± 0.2	2.8 ± 0.2^*^	2.6 ± 0.1^*^	3.5 ± 0.2
Butyric acid (mmol/kg)	Feces Week 6	15.9 ± 4.3	3.8 ± 0.9	8.7 ± 2.1	4.0 ± 0.8
	Feces Week 9	27.6 ± 6.9	1.6 ± 0.5^*^	1.2 ± 0.2^*^	1.8 ± 0.4^*^
	Cecal content	20.5 ± 1.2	15.9 ± 1.4	29.7 ± 1.7^*,**^	19.3 ± 1.1^***^
Isovaleric acid (mmol/kg)	Feces Week 6	2.3 ± 0.4	1.2 ± 0.3	0.5 ± 0.1^*^	0.7 ± 0.1^*^
	Feces Week 9	1.2 ± 0.1	0.3 ± 0.1^*^	0.1 ± 0.1^*^	0.3 ± 0.1^*,***^
	Cecal content	4.3 ± 0.2	2.6 ± 0.3^*^	2.0 ± 0.1^*^	3.5 ± 0.3^***^
Valeric acid (mmol/kg)	Feces Week 6	2.0 ± 0.4	0.7 ± 0.2	0.7 ± 0.2	0.7 ± 0.2
	Feces Week 9	1.4 ± 0.2	0.1 ± 0.1^*^	0.2 ± 0.1^*^	0.3 ± 0.1^*^
	Cecal content	3.4 ± 0.2	3.0 ± 0.3	3.0 ± 0.2	3.6 ± 0.3
Total SCFAs (mmol/kg)	Feces Week 6	223 ± 43	72 ± 11^*^	194 ± 33^**^	93 ± 20^*^
	Feces Week 9	180 ± 25	38 ± 6^*^	55 ± 9^*^	32 ± 3^*^
	Cecal content	168 ± 11	166 ± 11	212 ± 8^*,**^	169 ± 4^***^

*Note*: Values are expressed as means with their SEM. The number of animals per group is indicated in the table header. Total SCFAs refers to the sum of the SCFA analyzed (acetic, propionic, isobutyric, butyric, isovaleric, and valeric acids). *p* values were calculated using the one‐way ANOVA and the Tukey multiple‐comparisons test or by the nonparametric Kruskal–Wallis test and the Mann–Whitney *U* test. ^*^ *p* value ≤ 0.05 versus STD; ^**^ *p* value ≤ 0.05 versus HF; and ^***^ *p* value ≤ 0.05 versus HF+RS.

Abbreviations: HF, rats fed a high‐fat diet; HF+FG, rats fed a high‐fat diet with 0.1% of buckwheat d‐fagomine by feed weight; HF+RS, rats fed a high‐fat diet with 15% of high‐amylose maize resistant starch Type 2 by feed weight; SCFA, short‐chain fatty acid; STD, rats fed a standard diet.

### 3.6 Histology of Adipose Tissue and Liver

In PAT (Table 3), the HF group exhibited an increased total histological score compared to the STD group, despite showing no statistically significant differences in individual parameters, such as the presence of variable adipocyte diameter, lipoblastic vacuoles, mastocytes, septal fibrosis, angiomatous vascularization, and the grade of periadipocyte and septal histiocytes. In contrast, the HF+RS and HF+FG groups showed total histological scores similar to the STD group, indicating less histological alteration than that observed in the HF group.

**TABLE 3:** Categorization of histological parameters in perigonadal adipose tissue samples.

Item	Score	STD (*n* = 12)	HF (*n* = 12)	HF+RS (*n* = 11)	HF+FG (*n* = 12)
Variable adipocyte diameter (%)					
Absence	0	91.7	75.0	81.8	75
Presence	1	8.3	25.0	18.2	25
Lipoblastic vacuoles (%)					
Absence	0	100	91.7	90.9	100
Presence	1	0	8.3	9.1	0
Mastocytes (%)					
Absence	0	50.0	41.7	100	83.3
Presence	1	50.0	58.3	0	16.7
Septal fibrosis (%)					
Absence	0	75.0	66.7	36.4	50
Presence	1	25.0	33.3	63.6	50
Angiomatous vascularization (%)					
Absence	0	58.3	33.3	72.7	41.7
Presence	1	41.7	66.7	27.3	58.3
Focal mild inflammation (%)					
Absence	0	100	100	90.9	100
Presence	1	0	0	9.1	0
Grade of periadipocyte histiocytes (%)					
Absent	0	25.0	0	0	25
Mild	1	25.0	16.7	27.3	16.7
Moderate	2	16.7	41.7	27.3	33.3
Severe	3	33.3	41.7	45.5	25
Grade of septal histiocytes (%)					
Absent	0	100	83.3	100	91.7
Mild	1	0	16.7	0	8.3
Moderate	2	0	0	0	0
Severe	3	0	0	0	0
Total histological score	0–12	3 (2.25–4)	4 (3.25–4.75)^*^	3 (3‐4)	3 (2‐4.75)

*Note*: Values are expressed as frequencies (%) or median and 25th–75th percentiles. The number of animals per group is indicated in the table header. Total histological score was the sum of evaluated items. *p* values were calculated by means of contingency tables using *χ* ^2^ or by the nonparametric Kruskal–Wallis test and the Mann–Whitney *U* test. ^*^ *p* value < 0.05 versus STD.

Abbreviations: HF, rats fed a high‐fat diet; HF+FG, rats fed a high‐fat diet with 0.1% of buckwheat d‐fagomine by feed weight; HF+RS, rats fed a high‐fat diet with 15% of high‐amylose maize resistant starch Type 2 by feed weight; STD, rats fed a standard diet.

Regarding liver histology (Table 4), the HF and HF+FG groups presented increased liver steatosis compared to the STD group, particularly in the grade of steatosis. However, no significant differences were found in the total histological score across the groups. Notably, the HF+RS group showed a histological pattern similar to that found in the STD group, mainly in the characterization of liver steatosis. Additionally, all three HF groups showed a lower grade of portal chronic inflammation compared to the STD group.

**TABLE 4:** Categorization of histological parameters in liver samples.

Item	Score	STD (*n* = 12)	HF (*n* = 12)	HF+RS (*n* = 11)	HF+FG (*n* = 12)
Grade of steatosis (%)					^*^
Absence (<5%)	0	58.3	25.0	54.5	25.0
Mild (5%–33%)	1	41.7	50.0	36.4	50.0
Moderate (33%–66%)	2	0	25.0	9.1	25.0
Severe (>66%)	3	0	0	0	0
Steatosis localization (%)					
Absence	0	58.3	25	54.5	25
Centrilobular	1	0	0	0	0
Periportal	2	8.3	75	45.5	41.7
No zonal	3	0	0	0	0
Panacinar	4	33.3	0	0	33.3
Type of steatosis (%)					
Microvesicular steatosis	0–100	95 (95–95)	90 (90–95)	95 (95–95)	90 (70–95)
Macrovesicular steatosis	0–100	5 (5–5)	10 (5–10)	5 (5–5)	10 (5–30)
Lipogranuloma (%)					
Absence	0	100	91.7	90.9	58.3
Presence	1	0	8.3	9.1	41.7
Microgranuloma (%)					
Absence	0	8.3	8.3	18.2	100
Presence	1	91.7	91.7	81.8	0
Grade of portal chronic inflammation (%)				^*^	^*^
Absent	0	8.3	66.7	72.7	66.7
Mild	1	83.3	33.3	27.3	33.3
Moderate	2	8.3	0	0	0
Severe	3	0	0	0	0
Grade of sinusoidal dilatation (%)					
Absence	0	91.7	58.3	27.3	50
Mild	1	8.3	41.7	72.7	50
Severe	2	0	0	0	0
Grade of fibrosis (%)					
Absence	0	100	100	100	100
Portal fibrosis expansion	1	0	0	0	0
Incomplete porto‐portal or porto‐centrilobular fibrous bridges	2	0	0	0	0
Complete porto‐portal or porto‐centrilobular fibrous	3	0	0	0	0
Total histological score	0–10	2.5 (2–3)	3 (1.5–4)	2.5 (1–4)	3 (2–4)

Values are expressed as frequencies (%) or median and 25th–75th percentiles. The number of animals per group is indicated in the table header. Total histological score was the sum of the following evaluated items: grade of steatosis, presence of lipogranuloma, presence of microgranuloma, grade of portal chronic inflammation, and grade of sinusoidal dilatation. *p* values were calculated by means of contingency tables using *χ* ^2^ or by the nonparametric Kruskal–Wallis test and the Mann–Whitney *U* test. ^*^ *p* value < 0.05 versus STD.

Abbreviations: HF, rats fed a high‐fat diet; HF+FG, rats fed a high‐fat diet with 0.1% of buckwheat d‐fagomine by feed weight; HF+RS, rats fed a high‐fat diet with 15% of high‐amylose maize resistant starch Type 2 by feed weight; STD, rats fed a standard diet.

### 3.7 Oxidative Stress Parameters in Urine, Peritoneal Macrophages, Blood, and Tissues

During Weeks 5–6, the HF (*p* value = 0.066) and HF+FG (*p* value < 0.05) groups showed lower nitrites in urine than the STD group (Table 5). At the end of the study, the three HF groups presented an increase in plasma GSH compared to the STD group. The HF+RS and HF+FG groups had higher plasma GSSG than the STD group without relevant changes in GSSG/GSH ratio. Although differences in AUCs did not reach statistical significance among the groups, the HF+FG group presented the lowest production of intracellular ROS from peritoneal macrophages, being statistically significant compared to the HF and HF+RS groups at min 120 of the dichlorofluorescein assay (data not shown).

**TABLE 5:** Biomarkers of oxidative stress in urine, perigonadal macrophages, and blood.

	STD (*n* = 12)	HF (*n* = 12)	HF+RS (*n* = 11)	HF+FG (*n* = 12)
Urine (Weeks 5–6)				
Nitrite (µmol/L)	65 ± 8	42 ± 4	54 ± 8	32 ± 5^*^
Peritoneal macrophages				
ROS (AUC)	(69 ± 6) × 10^3^	(75 ± 8) × 10^3^	(73 ± 10) × 10^3^	(51 ± 9) × 10^3^
Plasma				
GSH (nmol/mL)	13 ± 1	17 ± 1^*^	17 ± 1^*^	21 ± 1^*^
GSSG (nmol/mL)	19 ± 1	22 ± 1	25 ± 1^*^	25 ± 1^*^
GSSG/GSH ratio	1.5 ± 0.1	1.4 ± 0.1	1.5 ± 0.1	1.2 ± 0.1
TBARS (nmol MDA Eq/mL)	2.2 ± 0.1	2.2 ± 0.1	2.0 ± 0.1	1.8 ± 0.1
Erythrocytes				
SOD (U/g Hb)	(2.93 ± 0.21) × 10^3^	(3.22 ± 0.15) × 10^3^	(2.61 ± 0.06) × 10^3^	(2.69 ± 0.08) × 10^3^
CAT (U/g Hb)	(29 ± 1) × 10^3^	(32 ± 1) × 10^3^	(33 ± 1) × 10^3^	(35 ± 1) × 10^3*^
GPx (U/g Hb)	72 ± 4	82 ± 4	86 ± 3	90 ± 3^*^
GR (U/g Hb)	1.2 ± 0.1	0.9 ± 0.1^*^	0.6 ± 0.1^*^	0.6 ± 0.1^*,**^
CAT/SOD ratio	10 ± 1	10 ± 1	13 ± 1^*,**^	13 ± 1^*,**^
SOD/GPx ratio	41 ± 2	40 ± 2	31 ± 1^*,**^	30 ± 1^*,**^
CAT/GPx ratio	414± 14	401± 13	388 ± 18	394 ± 11
GPx/GR ratio	58 ± 2	97 ± 6^*^	163 ± 38^*^	158 ± 17^*,**^
GSH (µmol/g Hb)	0.19 ± 0.04	0.40 ± 0.09	0.27 ± 0.03	0.27 ± 0.03
GSSG (µmol/g Hb)	1.2 ± 0.1	1.0 ± 0.1	1.0 ± 0.0	1.0 ± 0.1
GSSG/GSH ratio	8 ± 2	3 ± 1	5 ± 1	5 ± 1
TBARS (nmol MDA Eq/g Hb)	0.46 ± 0.06	0.63 ± 0.02	0.52 ± 0.12	0.57 ± 0.10

*Note*: Values are expressed as means with their SEM. The number of animals per group is indicated in the table header. *p* values were calculated using the one‐way ANOVA and the Tukey multiple‐comparisons test or by the nonparametric Kruskal–Wallis test and the Mann–Whitney *U* test. ^*^ *p* value ≤ 0.05 versus STD; ^**^ *p* value ≤ 0.05 versus HF; and ^***^ *p* value ≤ 0.05 versus HF+RS.

Abbreviations: AUC, area under the curve (fluorescence unit/2.5 × 10^4^ cells per 100 mL per 120 min); CAT, catalase; GPx, glutathione peroxidase; GR, glutathione reductase; GSH, reduced glutathione; GSSG, oxidized glutathione; Hb, hemoglobin; HF, rats fed a high‐fat diet; HF+FG, rats fed a high‐fat diet with 0.1% of buckwheat d‐fagomine by feed weight; HF+RS, rats fed a high‐fat diet with 15% of high‐amylose maize resistant starch Type 2 by feed weight; MDA Eq, malondialdehyde equivalent; SOD, superoxide dismutase; STD, rats fed a standard diet; TBARS, thiobarbituric acid‐reactive substances.

In erythrocytes, the three HF groups showed lower GR activity than the STD group (Table 5). The HF+RS (*p* value = 0.072) and HF+FG (*p* value < 0.05) groups also showed decreased GR activity compared to the HF group. Furthermore, the HF+RS (*p* value = 0.064) and HF+FG (*p* value < 0.05) groups showed increased GPx activity compared to the STD group. Overall, the HF+RS and HF+FG groups increased values of the GPx/GR ratio. The HF+RS and HF+FG groups similarly decreased SOD activity compared to the HF group, modulating ratios of CAT and GPx over SOD. The HF+FG group showed increased CAT activity compared to the STD group.

In PAT (Table 6), the HF and HF+FG groups showed lower TBARS than the STD group, whereas the HF+RS group showed attenuation of the decrease. Although differences in SOD activity did not reach statistical significance among the groups, the HF+RS and HF+FG groups similarly modulated the corresponding antioxidant enzyme ratios promoting CAT and GPx over SOD, especially evident after receiving FG that promoted higher CAT activity (CAT/SOD ratio, *p* value = 0.085 for HF+RS vs. HF groups and *p* value < 0.05 for HF+FG vs. HF groups).

**TABLE 6:** Biomarkers of oxidative stress in perigonadal adipose tissue.

Perigonadal adipose tissue	STD (*n* = 12)	HF (*n* = 12)	HF+RS (*n* = 11)	HF+FG (*n* = 12)
SOD (U/mg protein)	10.1 ± 1.4	9.8 ± 1.2	6.8 ± 0.8	8.3 ± 1.6
CAT (mU/g protein)	(3.9 ± 0.8) × 10^3^	(4.4 ± 0.5) × 10^3^	(5.0 ± 0.5) × 10^3^	(6.8 ± 1.0) × 10^3^
GPx (mU/g protein)	97 ± 9	101 ± 10	117 ± 13	97 ± 10
GR (mU/ mg protein)	52 ± 3	41± 4	44 ± 4	39 ± 3
CAT/SOD ratio	(0.33 ± 0.06) × 10^3^	(0.50 ± 0.08) × 10^3^	(0.85 ± 0.01) × 10^3*^	(0.88 ± 0.01) × 10^3*,**^
SOD/GPx ratio	4.4 ± 1.1	2.5 ± 0.4	1.4 ± 0.2^*^	1.4 ± 0.3^*^
CAT/GPx ratio	(53 ± 14) × 10^3^	(46 ± 6) × 10^3^	(49 ± 7) × 10^3^	(95 ± 30) × 10^3^
GPx/GR ratio	2.0 ± 0.3	2.6 ± 0.3	3.0 ± 0.5	2.7 ± 0.3
GSH (nmol/mg protein)	1.1± 0.2	1.4 ± 0.2	1.1 ± 0.1	0.7 ± 0.1
GSSG (nmol/mg protein)	4.4 ± 0.5	4.7 ± 0.4	3.9 ± 0.5	4.2 ± 0.7
GSSG/GSH ratio	4.4 ± 0.5	4.1 ± 0.6	4.4 ± 0.9	11.6 ± 3.9
TBARS (nmol MDA Eq/mg protein)	0.47 ± 0.08	0.21 ± 0.03^*^	0.28 ± 0.04	0.18 ± 0.05^*^

*Note*: Values are expressed as means with their SEM. The number of animals per group is indicated in the table header. *p* values were calculated using the one‐way ANOVA and the Tukey multiple‐comparisons test or by the non‐parametric Kruskal‐Wallis test. ^*^ *p* value ≤ 0.05 vsrsus STD; ^**^ *p* value ≤ 0.05 versus HF; and ^**^^*^ *p* value ≤ 0.05 versus HF+RS.

Abbreviations: CAT, catalase; GPx, glutathione peroxidase; GR, glutathione reductase; GSH, reduced glutathione; GSSG, oxidized glutathione; HF, rats fed a high‐fat diet; HF+FG, rats fed a high‐fat diet with 0.1% of buckwheat d‐fagomine by feed weight; HF+RS, rats fed a high‐fat diet with 15% of high‐amylose maize resistant starch Type 2 by feed weight; MDA Eq, malondialdehyde equivalent; SOD, superoxide dismutase; STD, rats fed a standard diet; TBARS, thiobarbituric acid‐reactive substances.

In the liver (Table 7), the three HF groups showed higher GPx activity than that observed in the STD group, affecting values of corresponding antioxidant ratios (i.e., SOD/GPx, CAT/GPx, and GPx/GR). The HF+FG group showed lower activation of GPx than the other two groups fed HF diets and tended to normalize CAT/GPx ratio (*p* value = 0.084 for HF+FG vs. STD groups; *p* value = 0.073 for HF+FG vs. HF groups) and GPx/GR ratios (*p* value = 0.07 for HF+FG vs. STD groups), but decreased the amount of GSH compared to the STD group. The HF+RS group attenuated the increase in GPx/GR ratio promoted by the HF diet (*p* value = 0.058 for HF+RS vs. STD groups).

**TABLE 7:** Biomarkers of oxidative stress in liver.

Liver	STD (*n* = 12)	HF (*n* = 12)	HF+RS (*n* = 11)	HF+FG (*n* = 12)
SOD (U/g tissue)	(6.0 ± 0.4) × 10^3^	(6.1 ± 0.4) × 10^3^	(6.0 ± 0.4) × 10^3^	(4.9 ± 0.3) × 10^3^
CAT (U/g tissue)	(7.3 ± 0.5) × 10^3^	(7.6 ± 0.3) × 10^3^	(7.5 ± 0.1) × 10^3^	(7.6 ± 0.2) × 10^3^
GPx (U/g tissue)	42 ± 1	59 ± 1^*^	57 ± 1^*^	51 ± 1^*,**,***^
GR (U/g tissue)	8.6 ± 0.7	7.5 ± 0.6	8.3 ± 0.7	7.7 ± 0.7
CAT/SOD ratio	1.3 ± 0.1	1.3 ± 0.1	1.3 ± 0.1	1.6 ± 0.1
SOD/GPx ratio	141 ± 10	103 ± 6^*^	106 ± 9^*^	95 ± 6^*^
CAT/GPx ratio	173 ± 11	130 ± 7^*^	132 ± 2^*^	149 ± 5^***^
GPx/GR ratio	5.2 ± 0.4	8.4 ± 0.6^*^	7.3 ± 0.6	7.2 ± 0.6
GSH (µmol/g tissue)	1.8 ± 0.1	1.6 ± 0.1	1.4 ± 0.2	1.3 ± 0.1^*^
GSSG (µmol/g tissue)	1.2 ± 0.1	1.2 ± 0.1	1.5 ± 0.2	1.3 ± 0.1
GSSG/GSH ratio	0.75 ± 0.11	0.79 ± 0.08	2.22 ± 1.02	1.09 ± 0.16
MDA + 4‐HAE (nmol MDA Eq/g tissue)	29 ± 2	26 ± 1	27 ± 2	28 ± 1

*Note*: Values are expressed as means with their SEM. The number of animals per group is indicated in the table header. *p* values were calculated using the one‐way ANOVA and the Tukey multiple‐comparisons test or by the non‐parametric Kruskal‐Wallis test. ^*^ *p* value ≤ 0.05 versus STD; ^**^ *p* value ≤ 0.05 versus HF; and ^***^ *p* value ≤ 0.05 versus HF+RS.

Abbreviations: CAT, catalase; GPx, glutathione peroxidase; GR, glutathione reductase; GSH, reduced glutathione; GSSG, oxidized glutathione; HF, rats fed a high‐fat diet; HF+FG, rats fed a high‐fat diet with 0.1% of buckwheat d‐fagomine by feed weight; HF+RS, rats fed a high‐fat diet with 15% of high‐amylose maize resistant starch Type 2 by feed weight; MDA Eq, malondialdehyde equivalent; SOD, superoxide dismutase; STD, rats fed a standard diet; TBARS, thiobarbituric acid‐reactive substances.

### 3.8 Liver Eicosanoids

The three HF groups showed decreased amount of eicosanoids compared to the STD group (Table 8). The HF+RS group exhibited intermediate levels, with a statistically significant increase in 20‐HETE compared to the HF and HF+FG groups. The 5‐HEPE/5‐HETE ratio remained consistent across all groups.

**TABLE 8:** Eicosanoids in liver at the end of the study.

	STD (*n* = 12)	HF (*n* = 12)	HF+RS (*n* = 11)	HF+FG (*n* = 12)
5‐HEPE (nmol/g tissue)	0.151 ± 0.017	0.086 ± 0.009^*^	0.105 ± 0.019^*^	0.094 ± 0.011^*^
11‐HEPE (nmol/g tissue)	0.30 ± 0.04	0.13 ± 0.01^*^	0.17 ± 0.03^*^	0.14 ± 0.02^*^
5‐HETE (nmol/g tissue)	2.0 ± 0.2	1.2 ± 0.1^*^	1.4 ± 0.2^*^	1.2 ± 0.1^*^
20‐HETE (nmol/g tissue)	0.145 ± 0.007	0.085 ± 0.008^*^	0.121 ± 0.011^*,**^	0.082 ± 0.008^*,***^
11(12)‐EET (nmol/g tissue)	0.22 ± 0.02	0.11 ± 0.01^*^	0.11 ± 0.01^*^	0.09 ± 0.01^*^
12‐HETE (nmol/g tissue)	2.02 ± 0.28	0.99 ± 0.15^*^	1.27 ± 0.25^*^	0.96 ± 0.13^*^
15‐HETE (nmol/g tissue)	0.80 ± 0.09	0.33 ± 0.04^*^	0.40 ± 0.07^*^	0.35 ± 0.04^*^
15‐HETrE (nmol/g tissue)	0.41 ± 0.03	0.41 ± 0.02	0.39 ± 0.03	0.38 ± 0.03
PGD_2_ (nmol/g tissue)	1.08 ± 0.13	0.32 ± 0.05^*^	0.50 ± 0.08^*^	0.38 ± 0.04^*^
PGE_2_ (nmol/g tissue)	0.63 ± 0.08	0.23 ± 0.04^*^	0.42 ± 0.08	0.29 ± 0.04^*^
Sum of HEPEs	0.45 ± 0.05	0.22 ± 0.02^*^	0.28 ± 0.05^*^	0.24 ± 0.03^*^
Sum of HETEs	4.9 ± 0.5	2.6 ± 0.3^*^	3.2 ± 0.5^*^	2.6 ± 0.3^*^
5‐HEPE/5‐HETE ratio	0.076 ± 0.005	0.072 ± 0.004	0.071 ± 0.003	0.081 ± 0.005
Sum of PGs	1.71 ± 0.20	0.55 ± 0.09^*^	0.92 ± 0.16^*^	0.67 ± 0.08^*^

Values are expressed as means with their SEM. The number of animals per group is indicated in the table header. Sum of HEPEs includes 5‐HEPE and 11‐HEPE. Sum of HETEs includes 5‐HETE, 12‐HETE, 15‐HETE, and 20‐HETE. Sum of PGs includes PGD_2_ and PGE_2_. *p* values were calculated using the one‐way ANOVA and the Tukey multiple‐comparisons test or by the nonparametric Kruskal‐Wallis test. ^*^ *p* value ≤ 0.05 versus STD; ^**^ *p* value ≤ 0.05 versus HF; and ^***^ *p* value ≤ 0.05 versus HF+RS.

Abbreviations: 5‐ETE, 5‐hydroxy‐6E,8Z,11Z,14Z‐eicosatetraenoic acid; 11(12)‐EET, 11(12)‐epoxy‐5Z,8Z,14Z‐eicosatrienoic acid; 5‐HEPE, 5‐hydroxy‐6E,8Z,11Z,14Z,17Z‐eicosapentaenoic acid; 11‐HEPE, 11‐hydroxy‐5Z,8Z,12E,14Z,17Z‐eicosapentaenoic acid; 12‐HETE, 12‐hydroxy‐5Z,8Z,10E,14Z‐eicosatetraenoic acid; 15‐HETE, 15‐hydroxy‐5Z,8Z,11Z,13E‐eicosatetraenoic acid; 20‐HETE, 20‐hydroxy‐5Z,8Z,11Z,14Z‐eicosatetraenoic acid; 15‐HETrE, 15‐hydroxyicosa‐8Z,11Z,13E‐trienoic acid; HF, rats fed a high‐fat diet; HF+FG, rats fed a high‐fat diet with 0.1% of buckwheat d‐fagomine by feed weight; HF+RS, rats fed a high‐fat diet with 15% of high‐amylose maize resistant starch Type 2 by feed weight; MDA Eq, malondialdehyde equivalent; PGD2, 9S,15S‐dihydroxy‐11‐oxo‐5Z,13E‐prostadienoic acid; PGE2, 9‐oxo‐11R,15S‐dihydroxy‐5Z,13E‐prostadienoic acid; STD, rats fed a standard diet.

## 4 Discussion

This study evaluated the preventive effects of RS (15%) and FG (0.1%) on prediabetes development in an HF diet model. As previously described [30], an HF, low‐fiber diet for 10 weeks induced obesity and prediabetes in male WKY rats. This prediabetic state is characterized by insulin resistance and compensatory hyperinsulinemia, through an increased number or size of pancreatic β‐cells for maintaining normal glucose levels [26, 48]. Body weight gain, glucose dysregulation, and increased plasma TAGs were already present by Weeks 4–6 under the HF diet. RS (15%) and FG (0.1%) reduced PAT weight gain, plasma TAGs, and TAGs/HDLc in plasma, but did not significantly improve insulin sensitivity. RS (15%) exerted greater beneficial effects on body weight gain, impaired glucose tolerance, HbA1c, and liver steatosis than FG (0.1%), likely due to its lower glycemic index and ability to modulate gut microbiota and SCFA production.

A meta‐analysis in humans has suggested inconsistent effects of RS on cardiometabolic risk factors [49]. In rats, RS at a dose of 27% by weight of feed may exert more benefits under STD than in HF conditions [50]. The TAG‐lowering effect of RS observed here aligns with previous findings in HF‐fed male rats [27], where RS (4%–16%) also reduced adipose tissue expansion. At a dose of 25%, RS improved insulin sensitivity without reducing abdominal fat in obese Zucker diabetic fatty (ZDF) rats [51]. Other studies, in male mice on an HF diet for 10 weeks, have shown no statistically significant effects on body weight, fat mass, glucose homeostasis, or TAGs with RS at a dose of 20% [52]. In contrast, in aged female mice on an HF diet for 16 weeks, the same dose (20%) prevented weight gain, liver steatosis, and inflammation [53].

As far as FG is concerned, its lack of effect on fasting glucose and insulin after 10 weeks aligns with prior studies in WKY rats under similar conditions [26]. FG (0.1%) benefits appear earlier (Weeks 6–10) under STD, reducing weight gain and inflammation [21] but require longer (Weeks 10–13) to improve glucose tolerance and inflammation under HF conditions [26]. In the present study, FG showed a tendency to attenuate impaired glucose tolerance by Week 8, indicated by a short plateau in OGTTs, but not at Week 4.

Metabolic disorders induced by the HF diet were accompanied by alterations in fecal microbiota and reduced the amount of SCFAs levels in feces and cecal content. RS, but not FG, modified microbiota, increased cecum weight, and increased SCFAs, indicating enhanced microbial activity by Week 6. These findings agree with previous studies [27, 50, 51, 52, 53]. Nevertheless, one of them did not find differences in the amount of SCFAs in cecal content [52]. Regarding microbiota, our results agree with a meta‐analysis showing reduced α‐diversity after RS intake without affecting β‐diversity [54], possibly due to enrichment of particular gut microorganisms that efficiently metabolize RS2 and/or its byproducts [55]. Other studies, in rodents, found differences in β‐diversity after receiving RS [51, 52], which could be in part related to increased doses of RS (20% in [52] and 25% in [51]). In the present study, RS not only reverted several alterations observed in fecal microbiota after 10 weeks on HF diet but also modulated others at phylum and genus levels, promoting the dominance of particular ones. In agreement with previous studies, RS decreased *Desulfovibrio* genus from the Proteobacteria phylum [52, 53]. Furthermore, it has been reported that consumption of high‐amylose RS2 can promote the growth of butyric acid‐generating bacteria of the phylum Firmicutes in mice [56] and increase the populations of *Ruminococcus bromii* and *Eubacterium rectale* in humans [57]. RS2 also promotes the growth of acetic‐ and propionic acid‐generating bacteria as suggested by in vitro studies [58, 59].

The beneficial effects of RS on metabolic health can be in part mediated by increased SCFA production (acetate, propionate, and butyrate) through microbial activity that can promote activation of adenosine monophosphate‐activated protein kinase (AMPK), a key regulator of energy metabolism. AMPK activation promotes fatty acid β‐oxidation, reduces adipose and hepatic lipogenesis [60, 61], resulting in decreasing fat mass and ectopic fat accumulation, and alleviating impaired glucose tolerance. In contrast, FG showed no effects on fecal microbiota, SCFA production, or metabolic health, differing from previous studies [20, 21, 26, 62]. These apparent discrepancies among studies may be explained by the animal model used (Sprague‐Dawley vs. WKY rats), duration of nutritional intervention (10 vs. 24 weeks) or feeding conditions (STD vs. HF diet).

It is known that dietary fat increases mitochondrial ROS production, leading to mitochondrial dysfunction and insulin resistance [63, 64]. In the present study, the HF diet slightly increased oxidative lipid damage in erythrocytes but not in liver or PAT, indicating that marked oxidative stress may take longer to observe during the progression of disease promoted by the HF diet for 10 weeks. Nevertheless, differences in oxidative damage to other biomolecules or specific subcellular localizations cannot be excluded. Previous studies have shown, in rats, that an HF high‐fructose diet for 20 weeks induces limited or inexistent oxidative stress of lipids while causing marked oxidative stress of proteins [65]. Here, prevention of lipid peroxidation in tissues may be, at least in part, achieved by general enhanced antioxidant response, especially evident in plasma GSH and liver GPx, which may be already present by Week 6 on the HF diet as evidenced by decreased urinary nitrites compared to the STD group. As previously observed, induction of a compensatory antioxidant response may be activated within 1 day upon HF diet for maintaining mitochondrial ROS at signaling levels [63]. Other studies have shown no increased mitochondrial production of ROS in the liver but increased production of those derived from NADPH oxidase (NOX) enzymes [65], which are particularly relevant in macrophages and in PAT. Nevertheless, we observed no differences in basal production of intracellular ROS from peritoneal macrophages between the HF and STD groups at the end of the study. In PAT, other authors also found no statistical differences in activities of antioxidant enzymes between HF‐fed mice and those mice fed a normal chow diet [66]. However, they observed an effective compensatory antioxidant response to elevated production of ROS associated with increased mitochondrial activity in brown adipose tissue of mice on HF diet for 20 weeks [66], highlighting the critical role of highly metabolic active tissues on early metabolic adaptations to HF diets.

The inclusion of RS or FG into the HF diet similarly modulated the activities of CAT and GPx over SOD in erythrocytes and PAT. In erythrocytes, the decrease in SOD observed may enhance hydrogen peroxide degradation, slowing the Fenton reaction and limiting hydroxyl radical formation in iron‐rich environments. This fact agrees with low values of lipid peroxidation by‐products and hemoglobin glycation in erythrocytes observed after receiving RS or FG compared to those found in HF controls. Regarding PAT, reduced SOD activity may increase the amount of superoxide anion radical derived from ROS‐producing enzymes such as NOX4. It has been previously described in retroperitoneal white adipose tissue of male mice fed an HF diet for 6 weeks, an increased expression of NOX4 together with an increase in GPx1 before the onset of obesity and insulin resistance [67]. Enhanced production of superoxide anion radical in inguinal white adipose tissue of mice through adipocyte‐specific deletion of mitochondrial SOD2 could potentiate mitochondrial biogenesis, lowering insulin resistance [68]. In the liver, RS and FG activated GPx at levels comparable to HF controls, preventing lipid peroxidation. However, elevated liver GPx, alongside fat accumulation, may contribute to insulin resistance, as ROS can positively regulate insulin signaling in HF‐fed animals [69]. Interestingly, FG exhibits the best results on the production of intracellular ROS from peritoneal macrophages. It is known that oxidative stress and inflammation are highly related. In previous long‐term studies, in rats, consumption of FG decreases the concentration of proinflammatory interleukin‐6 (already at Week 10 on HF diet) and n‐6 arachidonic acid‐derived eicosanoids in plasma of rats either on HF or STD diets for 24 weeks together with lower body weight gain and improved glucose homeostasis [21, 26], which may indicate modulation of immune response by FG. The mechanisms involved in these effects require further exploration.

The HF diet decreased the amount of eicosanoids in liver samples by the end of the study compared to the STD diet. Since eicosanoids are involved in inflammation and adhesion [6], these findings agree with those results from portal chronic inflammation observed by histological evaluation. Decreased amount of eicosanoids and lipid peroxidation by‐products after receiving the HF diet may be, in part, related to lower enrichment of PUFAs, together with higher amount of saturated and monounsaturated FAs in the liver and PAT compared to the STD diet as previously described [70, 71]. RS supplementation tended to restore eicosanoids (e.g., 20‐HETE, PGE2) to values closer to STD controls. A previous study has shown that RS increases the expression of several cytochrome P450 enzymes, including those involved in the production of n‐6 arachidonic acid‐derived 20‐HETE, in the liver of male mice on an HF diet, when compared to HF controls [52]. In particular, 20‐HETE has been involved in the regulation of blood pressure, inflammation, fatty acid β‐oxidation, and glucose homeostasis [72]. In the latter case, 20‐HETE can promote glucose‐stimulated insulin secretion by pancreatic β‐cells via interaction with free fatty acid receptor 1 (also referred to as GPR40) [73]. In addition to decreased liver steatosis, this effect could contribute to explaining why the OGTT was similar between the STD and HF+RS groups in the present study. Contrary, excessive amounts of 20‐HETE are linked to advanced diabetes progression in HF‐fed mice [74]. Furthermore, plasma 20‐HETE is increased in humans with obesity compared to their lean counterparts [75].

In conclusion, dietary consumption of RS (15%) or FG (0.1%) may delay the progression of metabolic disturbances promoted by an HF diet for 10 weeks, leading to animals being at least one step behind in the development of prediabetes compared to HF control ones. Beneficial effects were especially evident after receiving RS, which may be, in part, explained by its ability to modify gut microbiota and to enhance the production of SCFAs, indicative of the implication of gut microbiota in the onset and development of these metabolic disorders. Compared to RS, the lower effect of FG on these very early stages in the development of diabetes may be due to the low dose used, which corresponds to the minimum bioactive dose. The effect of a low dose of FG on perigonadal fat mass accumulation is similar to that of RS at a much higher dose. Other effects of FG may take longer to observe during the progression of disease promoted by the HF diet.

## Conflicts of Interest

The authors declare no conflicts of interest.

## Supporting information




**Supporting File**: mnfr70230‐sup‐0001‐SuppMat.docx.

## Data Availability

The data used in this study are stored on secure servers and are only accessible upon justified request and approval by the responsible researchers. For more information on data access, please contact the corresponding author.
