# Supplementary material for: Dietary Consumption of Type 2 Resistant Starch and d‐Fagomine Delays Progression of Metabolic Disturbances in Male Rats on High‐Fat Diet
Source: Mol Nutr Food Res. 2025 Sep 9;69(22):e70230. doi: 10.1002/mnfr.70230 (PMC12643193; doi:10.1002/mnfr.70230)
Supplement: Supplementary file 1 — Supporting File: mnfr70230‐sup‐0001‐SuppMat.docx. [file MNFR-69-e70230-s001.docx]

**SUPPLEMENTARY MATERIAL**

**Dietary Consumption of Type 2 Resistant Starch and d-Fagomine Delays Progression of Metabolic Disturbances in Male Rats on High-Fat Diet**

Bernat Miralles-Pérez^1,2^, Sara Ramos-Romero^3,4,5*^, María José Charpentier^1^, Vanessa Sánchez-Martos^1^, Àngels Fortuño-Mar^6^, Julia Ponomarenko^7,8^, Susana Amézqueta^9^, David Piñol-Piñol^3^, Xiang Zhang^10^, Josep Lluís Torres^3,5^, Marta Romeu^1*^

^1^ Facultat de Medicina i Ciències de la Salut, Universitat Rovira i Virgili, E-43201 Reus, Spain; MJC ORCID: 0009-0009-5698-5340, VSM ORCID: 0000-0002-6205-3509, MR ORCID: 0000-0002-2131-1858

^2^ Current: Instituto de Investigaciones Marinas-Consejo Superior de Investigaciones Científicas (IIM-CSIC), Eduardo Cabello 6, E-36208 Vigo, Spain; BMP ORCID: 0000-0003-1294-7069

^3^ Institute for Advanced Chemistry of Catalonia (IQAC-CSIC), E-08034 Barcelona, Spain; SRR ORCID: 0000-0002-9293-4454, DPP ORCID: 0000-0002-2511-1271, JLT ORCID: 0000-0002-5072-8265

^4^ Current: Department of Cell Biology, Physiology & Immunology, Faculty of Biology, University of Barcelona, E-08028 Barcelona, Spain

^5^ Nutrition & Food Safety Research Institute (INSA-UB), Maria de Maeztu Unit of Excellence, E-08921 Santa Coloma de Gramanet, Spain

^6^ Eldine Patología, C/ Plom 32, E-43006 Tarragona, Spain

^7^ Centre for Genomic Regulation (CRG), The Barcelona Institute of Science and Technology, E-08003 Barcelona, Spain; JP ORCID: 0000-0002-1477-9444

^8^ Universitat Pompeu Fabra (UPF), E-08003 Barcelona, Spain

^9^ Departament d’Enginyeria Química i Química Analítica, Universitat de Barcelona, E-08028 Barcelona, Spain; SA ORCID: 0000-0001-8976-467X

^10^ Department of Chemistry, University of Louisville, 2210 S. Brook Street, Louisville, KY E-40292, USA; XZ ORCID: 0000-0003-1102-6313

* Correspondence: marta.rome@urv.cat; Tel.: (+34) 977759378; sara.ramosromero@ub.edu; Tel.: (+34) 934021531

**Table S1.** Composition of the experimental diets

|  | **STD** | **HF** | **HF+RS** | **HF+FG** |
| --- | --- | --- | --- | --- |
| **Diet composition (g/kg feed)** | | | | |
| **Carbohydrate available** | **480** | **457** | **457** | **456** |
| Sucrose | – | 340 | 247 | 339 |
| Maltodextrin | – | 60 | 60 | 60 |
| Corn starch | – | 57 | – | 57 |
| Resistant corn starch | – | – | 150 | – |
| **Crude fibre** | **41** | **50^a^** | **50^a^** | **50^a^** |
| **Fat** | **40** | **230** | **230** | **230** |
| Soybean oil | – | 20 | 20 | 20 |
| Milk fat | – | 210 | 210 | 210 |
| **Protein** | **143** | **198** | **198** | **198** |
| Casein | – | 195 | 195 | 195 |
| L-Cystine | – | 3 | 3 | 3 |
| **d-fagomine** | – | – | – | **1** |
| Minerals | 28.4 | 43 | 43 | 43 |
| Vitamins | 1.2 | 19 | 19 | 19 |
| Ashes | 47 | – | – | – |
| Choline bitartrate | 1 | 3 | 3 | 3 |
| **Macronutrients (% caloric value)** | | | | |
| Energy from Carbohydrate (% kcal) | 67 | 39 | 37 | 39 |
| Energy from fat (% kcal) | 13 | 44 | 46 | 44 |
| Energy from protein (% kcal) | 20 | 17 | 18 | 17 |
| Total energy density (kcal/g) | 2.9 | 4.7 | 4.5 | 4.7 |

Abbreviations: STD, rats fed a standard diet (2014 Teklad Global 14% Protein chow from Inotiv); HF, rats fed a high-fat diet (TD.08811 45% kcal fat diet from Inotiv); HF+RS, rats fed a high-fat diet with 15% of high-amylose maize resistant starch type 2 by feed weight; HF+FG, rats fed a high-fat diet with 0.1% of buckwheat d-fagomine by feed weight. Energy intake was calculated by estimating metabolizable energy using the Atwater factors, assigning 4 kcal/g to protein, 9 kcal/g to fat, and 4 kcal/g to available carbohydrates except for RS, which used a factor of 2.8 kcal/g RS [1]. ^a^ Cellulose.

1. Tulley RT, Appel MJ, Enos TG, Hegsted M, McCutcheon KL, Zhou J, Raggio AM, Jeffcoat R, Birkett A, Martin RJ, Keenan MJ. Comparative methodologies for measuring metabolizable energy of various types of resistant high amylose corn starch. J Agric Food Chem. 2009,57(18):8474-8479. doi: 10.1021/jf900971c.

**Table S2.** Characteristics of the multiple reaction monitoring method for measurement of eicosanoids in the liver

| **Eicosanoid** | **Fatty acid / Pathway** | **Parent (m/z)** | **Daughter (m/z)** | **RT (min)** | **Cone voltage (V)** | **Collision voltage (V)** | **Dwell (s)** | **Internal standard** |
| --- | --- | --- | --- | --- | --- | --- | --- | --- |
| 5-HEPE | EPA / LOX | 317.0604 | 114.9811 | 6.09 | 2 | 12 | 0.025 | d_8_-15-HETE |
| 11-HEPE | EPA / NE | 317.1881 | 167.1318 | 5.86 | 6 | 16 | 0.025 | d_8_-15-HETE |
| 5-HETE | ARA / LOX | 319.2081 | 114.9700 | 6.58 | 8 | 14 | 0.025 | d_8_-15-HETE |
| 20-HETE | ARA / CYP | 319.2081 | 245.2898 | 5.83 | 8 | 14 | 0.025 | d_8_-15-HETE |
| 11(12)-EET | ARA / CYP | 319.3996 | 167.1216 | 6.83 | 2 | 12 | 0.025 | d_8_-15-HETE |
| 12-HETE | ARA / LOX | 319.3996 | 179.0961 | 6.38 | 10 | 12 | 0.025 | d_8_-15-HETE |
| 15-HETE | ARA / LOX | 319.3996 | 219.1805 | 6.19 | 2 | 12 | 0.025 | d_8_-15-HETE |
| 15-HETrE | DGLA / LOX | 321.2819 | 221.2607 | 6.42 | 6 | 14 | 0.025 | d_8_-15-HETE |
| PGD_2_ | ARA / COX | 351.1343 | 315.3484 | 3.84 | 2 | 8 | 0.025 | d_9_-PGD_2_ |
| PGE_2_ | ARA / COX | 351.1343 | 315.3484 | 3.72 | 2 | 8 | 0.025 | d_9_-PGD_2_ |
| d_9_-PGD_2_ | ARA / COX | 360.4043 | 324.2277 | 3.81 | 10 | 12 | 0.025 | d9-PGD2 |
| d_8_-15-HETE | ARA / LOX | 327.2128 | 226.1057 | 6.15 | 2 | 12 | 0.025 | d8-15-HETE |
| 15d-PGJ_2_ | ARA / COX | 315.1681 | 271.2903 | 5.82 | 2 | 10 | 0.025 | d_9_-PGD_2_ |
| 17(18)-EpETE | EPA / CYP | 317.1243 | 255.2942 | 6.21 | 8 | 10 | 0.025 | d_8_-15-HETE |
| 18-HEPE | EPA / CYP and NE | 317.1881 | 299.2679 | 5.67 | 4 | 8 | 0.025 | d_8_-15-HETE |
| LTB_4_ | ARA / LOX | 335.2319 | 195.1517 | 4.98 | 60 | 14 | 0.025 | d_9_-PGD_2_ |
| LxA_4_ | ARA / LOX | 351.2319 | 114.9115 | 4.17 | 26 | 12 | 0.025 | d_9_-PGD_2_ |
| PGD_1_ | DGLA / COX | 353.4634 | 317.3532 | 3.85 | 72 | 10 | 0.025 | d_9_-PGD_2_ |

Abbreviations: 5-HEPE, 5-hydroxy-6E,8Z,11Z,14Z,17Z-eicosapentaenoic acid; 11-HEPE, 11-hydroxy-5Z,8Z,12E,14Z,17Z-eicosapentaenoic acid; 5-HETE, 5-hydroxy-6E,8Z,11Z,14Z-eicosatetraenoic acid; 20-HETE, 20-hydroxy-5Z,8Z,11Z,14Z-eicosatetraenoic acid; 11(12)-EET, 11(12)-epoxy-5Z,8Z,14Z-eicosatrienoic acid; 12-HETE, 12-hydroxy-5Z,8Z,10E,14Z-eicosatetraenoic acid; 15-HETE, 15-hydroxy-5Z,8Z,11Z,13E-eicosatetraenoic acid; 15-HETrE, 15-hydroxyicosa-8Z,11Z,13E-trienoic acid; PGD_2_, 9S,15S-dihydroxy-11-oxo-5Z,13E-prostadienoic acid; PGE_2_, 9-oxo-11R,15S-dihydroxy-5Z,13E-prostadienoic acid; 15d-PGJ2, 11-oxo-5Z,9,12E,14E-prostatetraenoic acid; 17(18)-EpETE, 17(18)-epoxy-5Z,8Z,11Z,14Z-eicosatetraenoic acid; 18-HEPE, 18-hydroxy-5Z,8Z,11Z,14Z,16E-eicosapentaenoic acid; LTB_4_, 5S,12R-dihydroxy-6Z,8E,10E,14Z-eicosatetraenoic acid; LxA_4_, 5S,6R,15S-trihydroxy-7E,9E,11Z,13E-eicosatetraenoic acid; PGD_1_, 9S,15S-dihydroxy-11-oxo-13E-prostaenoic acid; EPA, eicosapentaenoic acid; ARA, arachidonic acid; DGLA, dihomo-γ-linolenic acid; LOX, lipoxygenase; NE, non-enzymatic; CYP, cytochrome P450; COX, cyclooxygenase; RT, retention time.
